# What helps older people persevere with yoga classes? A realist process evaluation of a COVID-19-affected yoga program for fall prevention

**DOI:** 10.1186/s12889-022-12818-5

**Published:** 2022-03-08

**Authors:** Abby Haynes, Heidi Gilchrist, Juliana S. Oliveira, Anne Grunseit, Catherine Sherrington, Stephen Lord, Anne Tiedemann

**Affiliations:** 1 Institute for Musculoskeletal Health, The University of Sydney and Sydney Local Health District, Level 10N, King George V Building, Royal Prince Alfred Hospital, Missenden Road, Camperdown, NSW 2050 Australia; 2 School of Public Health, Faculty of Medicine and Health, The University of Sydney, Edward Ford Building (A27) Fisher Road, Sydney, NSW 2006 Australia; 3 Prevention Research Collaboration, Sydney School of Public Health, Faculty of Medicine and Health, University of Sydney, Sydney, NSW 2006 Australia; 4 Falls, Balance and Injury Research Centre, Neuroscience Research Australia, UNSW, Sydney, NSW 2031 Australia; 5 School of Public Health and Community Medicine, Faculty of Medicine, UNSW, Sydney, 2052 Australia

**Keywords:** Realist evaluation, Yoga, Fall prevention, Healthy ageing, Telehealth, Intervention trial

## Abstract

**Background:**

Falls among older people are a major global health concern. This process evaluation investigates the experience of participants aged 60+ in a yoga program aimed at preventing falls which transitioned from studio-based classes to online classes in response to COVID-19 restrictions. We sought to understand how the Successful AGEing (SAGE) yoga program functioned in both settings and as a hybrid program, and to explain why it worked well for most participants.

**Methods:**

Realist process evaluation was used to explore the factors that facilitated a successful transition for most participants, and to consider why it did not work for a minority. This approach develops program theories that describe which mechanisms an intervention is (or is not) activating, and how this is mediated by context to generate process outcomes. Data included interviews with participants (*n* = 21) and yoga instructors (*n* = 3), self-report feedback forms (*n* = 46), observation of classes and routine process measures.

**Results:**

Factors that facilitated a successful transition for most participants included the quality of yoga instruction, the program format and inherent characteristics of yoga. Gains in transitioning online included continuity and greater convenience. Losses included perceived reduction in the effectiveness of yoga instruction. There were greater challenges for people struggling with pain and in disadvantageous home environments. We identified six program theories configured around 16 mechanisms: 1. It’s worth the effort and 2. In expert hands (these had the same mechanisms: value expectancy, therapeutic alliance and achievement/mastery), 3. A communal experience (these mechanisms were shared experience, social connection, social comparison and peer checking), 4. Putting yoga within reach (accessibility, convenience, gratitude), 5. Building yoga habits (purposeful structure, momentum, accountability and continuity), and 6. Yoga’s special properties (embodiment and mindfulness).

**Conclusions:**

This study showed that online delivery of a yoga program for people aged 60+ retained much of the value of a face-to-face program for the majority of participants, and increased the value for some. The structured, communal nature of an organised group program delivered by a skilled instructor, together with yoga’s intrinsic focus on mindfulness, facilitated continued engagement and perceived health benefits, despite the change in delivery mode.

**Supplementary Information:**

The online version contains supplementary material available at 10.1186/s12889-022-12818-5.

## Introduction

Falls among older people are a major global health concern with profound impacts on health and wellbeing. One in three people aged 65 and over fall every year, often causing serious injuries and ongoing disability [1]. Worldwide, falls are the second leading cause of death by accidental injury across all age groups [2] and the primary cause of injury-related death in people aged 70 and over [3]. Even in the absence of serious injury, fear of falling negatively impacts physical activity and quality of life [4, 5]. Falls also place a large burden on health systems and the economy due to an increasing proportion of older people in the global population [4, 6, 7].

### Yoga as a practise for healthy ageing

Exercise programs involving balance and functional activities are effective at preventing falls in older people [8]. Most forms of yoga challenge balance and provide improvements to stability and mobility [9] so have the potential to prevent falls. Yoga also promotes mindfulness which is associated with increased body awareness, agency and ownership [10]. Although there has been little research on the effect of yoga on falls [11], studies suggest that older people regard yoga as an acceptable and beneficial form of exercise, and an appropriate fall prevention strategy [12–14].

Yoga also offers many benefits for older adults. A recent meta-analysis and systematic review of the effects of yoga on people aged 60+ found significant positive outcomes compared with inactive controls in physical function (including balance and lower body flexibility), depression, perceived mental health, perceived physical health, sleep quality and vitality [15]. Yoga has also been shown to reduce anxiety [16], improve general quality of life [17] and satisfaction with life [18], support cognitive functioning [19] and assist in recovery from trauma or loss [20].

Increasingly, yoga is used in the management of specific health conditions that affect many older people. These include chronic obstructive pulmonary disease [21], cardiovascular disease and metabolic syndrome [22], rheumatoid arthritis [23], osteoarthritis [24], stroke [25] and different forms of musculoskeletal pain [26–29]. Yoga can also uniquely affect people’s physical self-regard, facilitating positive body image and self-compassion, and shifting focus from external physicality to more functional or internal aspects of the body. Such improvements have been shown to bolster intrinsic motivation for physical activity [30–32].

A number of studies suggest that yoga may be superior to conventional physical activity programs (such as aerobic exercise) in improving health-related outcomes for older people [33]. Traditional yoga practise has been found to be as safe, or safer, than forms of exercise such as cardiovascular fitness activities, running, soccer and tennis, especially if practised with supervision [34]. A longitudinal study of 9151 women aged 62–67 identified no adverse association between yoga practise and joint problems [35].

The global shift from face-to-face to online programs and services as a means of increasing reach and reducing costs, supported by technological innovation and widespread access to technology, has been accelerated by the COVID-19 pandemic. Telehealth can widen access for many, including people who have poor mobility, lack of transport options, caring roles or live outside of service areas [36]. Teleyoga (real-time yoga classes provided via interactive videoconferencing [29]) is a promising way of delivering yoga classes at scale, potentially reducing costs and increasing convenience, and reaching people who cannot attend studio-based classes, including those affected by COVID-19 restrictions [37]. Teleyoga has been used effectively in diverse populations, including with older people [38–42].

### The successful AGEing (SAGE) yoga trial

The Successful AGEing (SAGE) yoga trial is a randomised controlled trial testing the effect of a yoga-based exercise program on the primary outcome of falls among 700 community-dwelling people aged 60+ years. To our knowledge, SAGE is the first trial of this size to test the impact of yoga on falls. Participants are randomised to either: (1) the SAGE yoga program involving 40 weeks of supervised twice-weekly yoga-based exercise designed to prevent falls with the availability of flexible ‘make up’ classes, plus goal-setting and unsupervised homework using specially designed yoga resources, or (2) a seated yoga relaxation program taught over two group-based workshops and then undertaken individually as often as participants choose. Trial participants are recruited via local newspapers, community centres and through social media. The primary outcome is rate of falls in the 12 months following randomisation, measured on a monthly basis via self-report. Secondary outcomes include mental well-being, physical activity, health-related quality of life, balance self-confidence, physical function, pain, goal attainment and sleep quality. An economic analysis will compare the cost-effectiveness and cost-utility of the two yoga programs. Details are available elsewhere [6].

The SAGE program was designed to be delivered face-to-face in yoga studios but, following public health restrictions on movement in response to the COVID-19 pandemic, classes were moved online. For four classes, this change resulted in a ‘hybrid’ studio/online program with all but three of the 46 participants making the transition to online classes. Of those who continued (*n* = 43) 39 completed the full program via Zoom. Participants in the four hybrid classes experienced a wide range of ratios of studio-based to online sessions as those who started earliest attended 17 weeks of studio classes before COVID-19 struck whereas later classes met in the studio for only a few weeks. Most participants who persevered with the program rated it highly and reported positive health impacts, habit formation and intention to continue yoga.

### Study aims

The current study sought to understand how SAGE functioned as both a studio-based program and an online program, and to explain why it worked well for the majority of participants as a hybrid program. Our research questions were: In the SAGE yoga program, what is working, for whom, and under what circumstances? What aided the transition from studio-based to online yoga classes?

## Methods

### Study design

Given our explanatory research aims, we chose a realist process evaluation because of its utility for surfacing and testing program theories [43, 44]. Process evaluation seeks to shed light on why an intervention was or was not effective, and how it can be improved for better contextual fit [45]. The realist approach develops program theories that describe which mechanisms an intervention is (or is not) activating, and how this is mediated by contextual factors to generate outcomes [46, 47]. The realist approach is particularly useful for novel interventions such as SAGE where causal pathways are not yet well understood. Data driven theories explaining how programs work can strengthen intervention design, adaptation and evaluation; hone local implementation processes; and improve how interventions are targeted [45, 48]. Lastly, realist process evaluation uses mixed methods with an emphasis on qualitative exploration of participants’ causal explanations. Qualitative research is especially useful for improving trial recruitment, fine-tuning implementation and outcome measurement instruments, and explaining how the intervention functioned in relation to various contextual factors, thereby facilitating the transferability of findings to other settings [49–51].

### Conduct of the realist process evaluation

**Fig. 1:**
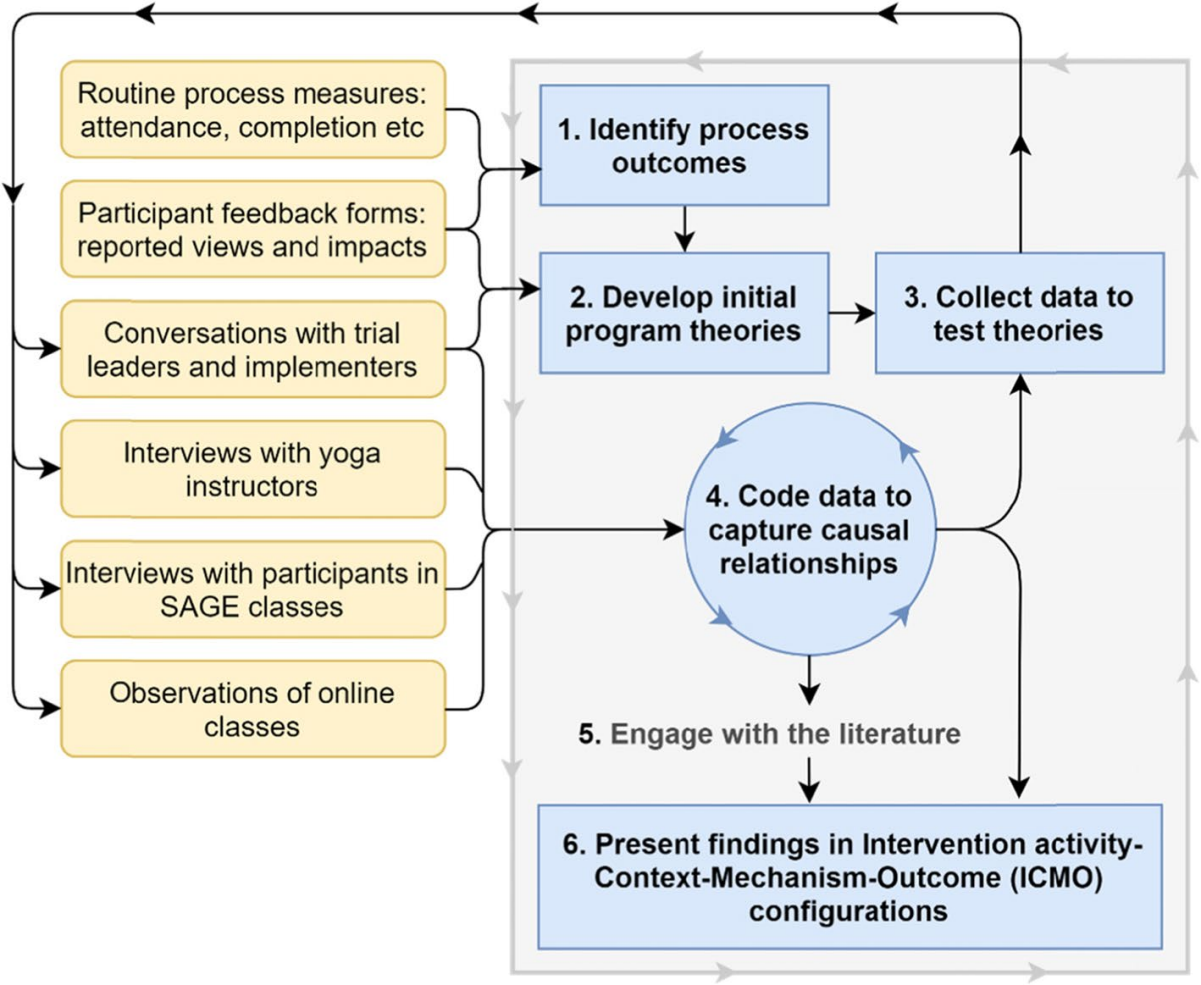
Overview of the SAGE process evaluation analytic process

We undertook six key tasks for this realist process evaluation, as illustrated in Fig. 1 and described below. These tasks have been described in more detail elsewhere [52].

#### Identify process outcomes

We identified three process outcomes: 1. Engagement with the yoga program, 2. Self-reported health impacts, and 3. Intention to continue yoga. These were informed by routine process measures, which indicated high levels of program completion, and post-intervention feedback data showing 87% of participants in the SAGE classes that transitions online reported some degree of improved physical or mental health, 78% reported improved balance, and 57% intended to continue with yoga (20% were unsure). Qualitative data included comments about improved flexibility, mobility, relaxation and/or sleep quality, and appreciation of/enjoyment in the program. Two further process outcomes were identified as data collection progressed and were refined with guidance from the literature: Habit formation [53, 54] and Physical literacy [55–57].

#### Develop initial program theories

Initial theories (Table 1) about how these outcomes were generated were developed via two sources: (a) discussion with the SAGE trial leaders and implementers about their underlying causal hypotheses, and (b) the literature on falls and older people’s engagement in yoga and yoga-based exercise, and physical activity more broadly. Our hypothesis was that the mechanisms identified in these theories were activated in both studio-based classes and online classes, hence the high rates of program continuation despite the disruption and challenges of transition.

**Table 1:** Initial program theories about how SAGE works for most participants

Initial program theory		Supporting theories from the literature
1.	People persevere with the program because they feel health benefits (e.g. improved balance and mobility) which boost feelings of strength and independence	PA generates feelings of physical and psychological wellness [58], and yoga does this particularly effectively, including for older people [33, 59, 60]
2.	The perceived quality of yoga instructors is important for making people feel safe and confident. This includes instructors who understand the needs of older people	The quality of instructors affects older people’s feelings of safety and confidence in PA [5]. The concept of therapeutic alliance is key to understanding instructor/participant relationship quality [61]
3.	Tailoring of the classes is crucial. People must feel they can participate according to their abilities and health needs	Tailoring of PA programs is a key motivator in adherence for older people [5, 62, 63]. Fall prevention interventions should be tailored for targeted recipients to maximise safety and effects [64]
4.	Group classes facilitate social connections which may add to the enjoyment of classes	Many empirical studies [62, 65–68] and theories back this concept including self-determination theory [69] and the upward spiral theory of lifestyle change [70]
5.	Free classes are an incentive to give yoga a go and stick with it long-term	Subsidised costs increase participation in PA [71, 72]. The ‘zero price effect’ [73] increases perceptions of value

#### Collect data to test these theories

Data collection aimed to test the initial program theories, which described hypotheses about the program-as-intended, by exploring them in the light of people’s experiences of the program-as-delivered. We conducted semi-structured phone interviews with all available participants in the hybrid program (*n* = 21), and with the current yoga instructors (*n* = 3). Interviewees were asked about their experiences of the hybrid program and any explanations for program impacts. We described our program theories at the end of each interview and asked interviewees to critique them. Observations of classes with different instructors were conducted to better understand implementation fidelity (were participants in different classes experiencing the same intervention? The same mechanisms?). Emails and texts from participants who withdrew during the transition period were also reviewed along with written comments in routine participant feedback forms (*n* = 46).

Interview duration ranged from 29 to 66 min with an average of 50 min. Fifteen trial participants were female (71%), reflecting the gender ratio in the trial, and aged between 61 and 80 with an average age of 68. This sample included four participants who stopped attending online classes, those who gave negative survey feedback and those with painful physical conditions such as osteoarthritis and scoliosis. All 3 yoga instructors were female with over 15 years’ experience of delivering Iyengar yoga.

#### Code data to capture causal relationships

Analysis was conducted in parallel with data collection. We imported interview transcripts into NVivo 12 [74] and coded deductively to our existing program theories, and inductively as we identified potential new theories. Data were double-coded for context, mechanisms and process outcomes where likely candidates were observed. Data in which interviewees explicitly compared the two modes of delivery were coded to a discrete node (Gains and Losses) in addition to any relevant theory nodes to facilitate comparative reasoning about what worked or not.

Two researchers (AH and HG) coded a proportion of transcripts independently using a preliminary coding frame, frequently discussing how newer transcripts were reshaping the existing program theories and/or indicating others. As interviews and their analysis progressed our theories evolved: two initial theories were merged, one was expanded and three theories were added to the final coding frame. Periodic workshops were held with co-authors acting as “critical friends” to review the unfolding analysis [75].

#### Engage with the literature

Initial program theories were stored in NVivo in a table with additional rows for theories developed inductively, and columns for notes from literature searches, data sources and revisions to track how they were evolving. We investigated initial theories and, later, our emergent theories in the literature to fine-tune them and to identify corroborating or disconfirming findings from other studies. We used likely keywords in Google Scholar and opportunistically followed citations of theoretical or empirical studies that suggested plausible explanations for our findings. Our search strategy was an iterative approach conducted throughout tasks 2, 3 and 4 where ‘clues’ about mechanisms suggested by the program designers and our interviewees were further investigated.

#### Present findings in ICMO configurations

Realist evaluation standards dictate that findings show connections between elements in the program theory [76]. We distilled our coded theories into a summary table of Intervention activity-Context-Mechanism-Outcome configurations (ICMOs) [46, 77] and used subheadings to make the table more accessible to readers unfamiliar with realist approaches (Table 2).

To strengthen research rigour, we used Ronkainen and Whiltshire’s framework for validity in realist research in sports and exercise psychology [75] (Additional file 1) and adhered to the RAMESES reporting standards for realist evaluations [76].

### Ethical approvals and consent

Ethical approval for this study was provided by the University of Sydney Human Research Ethics Committee, reference 2019/604. Interviewees gave prospective informed consent to take part in an interview at the trial commencement and confirmed this in their post-trial feedback form. They also gave verbal consent to the trial administrators for their details to be passed on to the interviewer, and confirmed this consent in the interview itself after information about audio recording and use of de-identified data had been stated.

## Results

Table 2 presents an overview of our final program theories showing the relationships between intervention activities, context and mechanisms. This addresses our first research question: In the SAGE yoga trial, what is working, for whom, and under what circumstances? We then describe these program theories in greater detail, using illustrative quotes and identifying supporting literature.

**Table 2:** An overview of how the SAGE yoga program worked for participants who took part in the hybrid program

Program theories		Intervention activities What did we do?	Context Who did it work for?	Mechanisms How did it work?	Outcomes With what process effects?
1.	It’s worth the effort	Program of yoga-based exercise with progressively challenging poses designed to prevent falls in older people	∙ The program attracted people who believed in the efficacy of yoga and who had interests in healthy ageing &/or tackling fall-related physical decline ∙ It best suited those with physical capabilities in the moderate range who had manageable levels of pain	∙ Value expectancy ∙ Therapeutic alliance ∙ Achievement/Mastery	∙ Engagement with SAGE: attendance (including across transition to online classes), expressed commitment and enjoyment ∙ Self-reported improvements in balance, flexibility, strength, mobility, stress reduction, sleep quality and/or wellbeing ∙ Habit formation: routine practise of yoga as part of everyday life ∙ Physical literacy: physical competence and confidence (self-efficacy), including the creation of transferable skills, and motivation to engage in physical activity ∙ Intention to continue yoga (or strength-based physical activity)
2.	In expert hands	Experienced instructors deliver the program, individualising it for participants’ different capabilities			
3.	A communal experience	Group classes with a maximum of 18 people WhatsApp forum for each group when classes moved online	∙ Group classes worked for those who valued social interaction &/or shared experiences ∙ Studio classes suited those who liked to benchmark their physical competence and/or peer-audit their poses	∙ Shared experience ∙ Social connection ∙ Social comparison ∙ Peer checking	
4.	Putting yoga within reach	Free classes in local yoga studios... ... then online via Zoom with tech support from the SAGE team	∙ Studio classes suited those with easy access to a participating yoga studio ∙ Online classes worked for those with suitable home environments and tech confidence or with hesitancy but openness to trying online classes with support	∙ Accessibility ∙ Convenience ∙ Gratitude	
5.	Building yoga habits	Twice-weekly classes over 12 months with: ∙ flexible ‘make up’ classes ∙ program-specific homework tools and encouragement ∙ goal-setting for mobility	∙ SAGE’s structure worked for people who prioritised and could commit to the schedule ∙ Homework suited those keenest on progression &/or their instructor’s approval ∙ Flexible classes were used by those with carer commitments, travel plans, injury or illness ∙ Goal-setting did not seem to work in SAGE	∙ Purposeful structure ∙ Momentum ∙ Accountability ∙ Continuity	
6.	Yoga's special properties	The SAGE program utilises core Iyengar yoga practices	∙ This worked best for those who were open to yoga as an holistic practise	∙ Embodiment ∙ Mindfulness	

### Program theory 1. It’s worth the effort

Our first program theory explains how people made cost/benefit analyses about the program, deciding to enrol and then persevere with the program based on its perceived value. SAGE appealed to people with interests in healthy ageing and/or in tackling fall-related physical decline so, initially, its value hinged on anticipated health benefits, a mechanism that was supported by contextual factors such as prior experience of yoga or yoga’s “good PR”. Value expectancy was boosted when physical and/or psychological health benefits were actually experienced, so for many participants the effort they put into yoga practise was well rewarded. Value assessments were attributed to multiple aspects of the program including: the intrinsic qualities of yoga (see program theory 6), the teaching strategies used by instructors to build skills and encourage progress (see program theory 2), cost and accessibility factors (program theory 4), the structure of SAGE (see program theory 5), and enhanced by the promise of a program that was designed especially for older people,

“It was important that it was framed as seniors’ yoga … that was a selling point for me because I thought, ‘Oh good…. It will be people in my own demographic…. I'm not going to be lying next to someone who’s thin and gorgeous and can, you know, put her right toe in her left ear’” (P4)

However, the level at which the yoga program was pitched was not optimal for everyone, despite their efforts. SAGE was best suited to those with physical capabilities in the moderate range who could practise in the ‘goldilocks zone’ of feeling challenged by the program yet able to meet that challenge. While a few of the younger more active participants would have liked greater challenge, some with debilitating physical conditions such as scoliosis found yoga too painful, even with modified poses. Two women who described themselves as very overweight felt some poses did not work well for their body shape.

This theory was supported by literature which indicates that older people’s engagement with physical activity can generate feelings of physical and psychological wellness [58, 63, 78] and that yoga [33, 59] (including teleyoga [60]) does this particularly effectively [33, 59]. The health belief model suggests that people experience value-expectancy when engaged in preventive health action where benefits seem to outweigh costs [79, 80]. The concept of early wins building a sense of achievement is supported by the concept of self-efficacy [81] and a realist evaluation of a yoga program in which participants’ realisation that “my body can do this” was identified as a mechanism [82].

### Program theory 2. In expert hands

In addition to the overall design of the program, the quality of yoga instructors was an essential ingredient to the appeal of the program. Key instructor attributes were: credibility as a yoga practitioner; empathy and other interpersonal skills; and excellent teaching skills including the ability to lead a whole class while simultaneously attending to the needs of individuals,“that’s why I think [Instructor 1] was such a good teacher because she never gave you a feeling that your different ability was an impediment, and she helped everyone at all levels” (P1).

“... she was an absolute gem, an exceptional teacher, warm, welcoming, practical, encouraging as well as highly competent in her field.... she's able to be so flexible in her approach [because] she has a toolkit at her disposal that she can deploy as needed.” (P5)

Many mentioned specific teaching techniques such as encouraging people to work to their own limits, checking in with individuals (e.g. asking about the state of an injured knee), and highlighting the group’s progress, for example, by revisiting a pose towards the end of the class so participants could see in-class improvements.

This program theory is supported by adult learning [83] and coaching theories [84], self-determination theory and its emphasis on autonomy support [58, 69], and the literature on older people, physical activity and falls (especially with regard to tailoring as a key motivator for older people) [5, 62–64]. Also, the concept of therapeutic alliance: an empowering relationship dynamic between health professionals and service users which is characterised by mutual respect, trust, negotiated task- and goal-setting, encouragement and support [16, 61, 85, 86].

### Program theory 3. A communal experience

This program theory was initially about social interaction (which was an incentive for a minority) but evolved to focus on the concept of shared experience or solidarity which enhanced the experience of yoga practise for all but one of our interviewees, regardless of how much they wanted to interact with the other group members, “[It’s] the feel of having other people around you, the feel of working together” (P14). When in the studio, the group setting also supported two other mechanisms: many interviewees said they liked to benchmark or compare their physical functioning against others in their peers, and others described checking the person next to them to see if they were interpreting an instruction correctly. Outside of classes the WhatsApp forum was used to advise instructors about absences but was seldom used for other social interaction, suggesting that the incidental nature of conversation enjoyed by many in the studio classes was not well served by this platform. The exception was one group who arranged a get-together after the program had finished. They were in the class with the longest pre- COVID-19 studio time (17 weeks), suggesting that increased face-to-face contact creates stronger social bonds which may be maintained when classes move online. However, interviewees mentioned this as a pleasant outcome of SAGE rather than a factor in their perseverance with the program.

Our theory of communal experience is supported by: the upward spiral theory of lifestyle change (in which perceptions of social integration in physical activity are associated with increased positive thoughts and feelings about that activity) [70], self-determination theory (the importance of satisfying relatedness needs) [58, 69], and the literature on social comparison [87, 88] and social factors that positively influence older people’s engagement with physical activity [62, 65–68, 89, 90].

### Program theory 4. Putting yoga within reach

This program theory focuses on the accessibility and convenience of SAGE classes, both studio-based and online. Free classes were appreciated by all interviewees and acted as an enrolment incentive for those who were unsure about the value of yoga. For some on pensions or low incomes, free classes put quality yoga instruction within their reach. Many expressed gratitude about this, “... I feel terribly lucky to have it” (P21) which extended to the physical location of participating yoga studios being close to interviewees homes. Online classes increased convenience substantially for most, but many participants (and instructors) initially struggled with technology. Transition was smoothest for those who were accustomed to using technology and felt confident in navigating its challenges, but it also worked for those who were inexperienced but open to trying online classes with support from the SAGE team or their family members. All interviewees had prior access to appropriate devices so there was minimal indication of digital inequities, but one said she struggled with the costs of maintaining connectivity.

This program theory was supported by the ‘zero price effect’ on value expectancy [73], studies of the impact of affordability on physical activity [71] (including for older people [5, 72]) and literature on older people’s use of technology for health and wellbeing [91–93].

### Program theory 5. Building yoga habits

The structure of the SAGE program supported participants to build and embed yoga as a habit. Interviewees were adamant that twice-weekly classes were essential for maximising health benefits but also explained that the regular schedule helped to shape their week (especially for retirees) and created a sense of momentum, “Once a week it starts to disappear out of your head and then you start again, whereas twice a week, you’re in this routine” (P16). However, this relied on willingness to prioritise SAGE and sufficient flexibility to do so (requiring family support in some cases). Flexible ‘make up’ classes were valued by interviewees with carer commitments, travel plans, or other unforeseen demands on their time, and were frequently used to catch up on classes missed due to injury or illness. SAGE’s homework tools (program specific videos and handouts) and encouragement by instructors to do homework seemed to work best for those who were keenest on progressing and/or liked their instructor’s approval. The latter worked because instructors made it clear they could tell who was doing homework which some participants found “... highly motivating!” (P5). This form of accountability was seen to motivate routine practise and “then you eventually become self-accountable” (P7). This theory is supported by habit formation theories [53, 54], the concept of autonomous accountability [94], the literature on healthy ageing [95] and rehabilitation theory [96].

### Program theory 6. Yoga’s special properties

The SAGE program is balance-focused but utilises core Iyengar yoga practices including sequences that focus on precise bodily alignment using poses (asanas), breathwork and the use of props (e.g., blocks, bolsters, straps). Interviewees described being uniquely engaged with these practices in a way that they did not associate with other forms of exercise or recreation. They found yoga to be calming and “almost meditational” (P1), but also “stimulating and energising” (P18). The literature offered several plausible explanatory (and complementary) theories. Embodiment theory [18, 31, 97, 98] states that yoga has a positive impact on dimensions of embodiment including: feeling ‘at home’ in one’s body, agency and functionality, and inwardly attuned self-care. These concepts aligned well with our data in which interviewees told us about focusing on their bodies, “I think that there is nothing to experience except your body and that doesn’t happen very often in modern society”, (P10) and noted they were working with their bodies rather than on their bodies. This practise built physical literacy [55–57] that interviewees were using in other parts of their lives, “I’ve hurt my back. I’ve just got a pulled muscle or something, and you know what I’m doing? My yoga stretches” (P17).

This was aided by mindfulness, “You get into a posture with your mind. Your mind helps your body get there” (P12). Mindfulness was strongly supported as a mechanism in the complementary health and neuroscience literature [32, 99–103]. Although few participants raised the concept of mindfulness (e.g. describing the program as “a process-oriented practise that brings you back to what’s happening in the present moment” (P14)), when prompted, all but one agreed that mindfulness was a factor in their engagement with SAGE. For some, it had built transferable stress reduction skills, “Mentally I think it helped me heaps because I really did use a lot of the yoga mindfulness ... through some pretty rough patches” (P4). Mindfulness was regarded by the instructors as being at the heart of the program, “Yoga is mindfulness. It’s asking you to stop. It’s asking you to be in your body. It’s asking you to be present” (I1). This was seen to bolster the program’s fall prevention goals,

“... often as we get older, we come to be much more in our head and we almost disconnect from our physical bodies and I think that’s why so many people fall, because they’re not really in their bodies anymore as much, especially in the legs. And so, with yoga and the way the program is being set out, it’s very much legwork orientated ... so it is forcing them to bring that awareness into their legs.” (I2)

Spirituality was highlighted in some studies of yoga, particularly in the US [104–106] literature, but very few interviewees raised this topic or felt it resonated with them when we prompted them in later interviews, possibly reflecting Australia’s more secular-leaning culture. Instructors told us they avoided the term ‘spiritual’ because it could be alienating. Even so, two interviewees suggested the program went too far in that direction, “I like yoga’s physicality. I don’t like the mumbo jumbo bits that come with it” (P20) and another reported a friend’s concern at yoga’s “anti-Christian” use of Hindu philosophy.

Observations suggested that classes run by different yoga teachers were very similar in content and differed only slightly in presentation style. No discernible difference between the classes run by different yoga instructors was evident in the interviewee data.

### Gains and losses: how did the hybrid program function?

This section provides an overview of what worked and what didn’t in the hybrid program that resulted from transition to online classes (Table 3) and addresses our second research question— What aided the transition from studio-based to online yoga classes?—focusing on gains and losses. It highlights that mechanisms functioned in different ways, and to varying extents, across the different modes of delivery in this hybrid program. For example, online classes offer greater accessibility and convenience, and retain the purposeful structure of studio-based classes but, for some, diminish therapeutic alliance, social connection, social comparison, and expectations regarding the effectiveness, and thus the value, of the program.

**Table 3:** Gains and losses: how did the hybrid program function?^a^

Intervention activities What did we do?	Context Who did it work for?	Mechanisms How did it work?
Our response to COVID-19 transformed SAGE from a studio-based yoga program to a hybrid studio/online program Attempts to minimise the impact of social restrictions included offering online yoga classes with the same group and instructor, 1:1 check-ins with instructors, tech support and a WhatsApp forum for each group	∙ COVID-19 restrictions impacted everyone. For some, restrictions made SAGE’s accessibility and purposeful structure more valuable ∙ The hybrid program worked best for those who: a. Believed that online instruction was effective. This was aided by embedding yoga fundamentals and forming trust in instructors during early studio classes b. Had conducive home environments with sufficient space and lack of distraction ∙ Muted classes aided focus but impeded communication so worked best for people who didn’t want/need to ask questions during classes ∙ The online platform prevented incidental social chat, disadvantaging those who liked conversation, but camaraderie established in studio classes held ∙ Poor visibility in online classes disadvantaged those who liked to benchmark physical competence or peer check poses	∙ Accessibility + ∙ Convenience + ∙ Purposeful structure + ∙ Continuity + ∙ Value expectancy +/− ∙ Embodiment +/− ∙ Mindfulness +/− ∙ Therapeutic alliance +/− ∙ Achievement/Mastery +/− ∙ Gratitude +/− ∙ Momentum +/− ∙ Accountability +/− ∙ Shared experience +/− ∙ Social connection - ∙ Social comparison - ∙ Peer checking -

^a^Outcomes for Gains and losses are the same as those shown above in Table 2

COVID-19 social restrictions impacted all participants and while they created losses in SAGE, they also made the online program’s accessibility and purposeful structure more valuable, and probably enhanced engagement for many, “Most of us really felt quite grateful that we could still do it online” (P12). Nevertheless, in feedback form responses 57% (*n* = 26) of participants said they preferred face-to-face classes, with 15% preferring online classes and 15% liking both equally (albeit for different reasons). Participants who struggled with online classes were those who felt disadvantaged by their home environment, e.g. having insufficient space or disruption from family members. A few expressed discomfort at the “intrusive” (P15) nature of videoconferencing bringing people into their home and several retirees living alone regretted the loss of the yoga studio as a calm, dedicated space that gave them a reason to leave their home.

Over half our interviewees felt that the online interface impeded the quality of observation by their instructor, leading to less effective instruction (but, importantly, not unsafe instruction). This concern was assuaged by instructors giving individualised feedback which convinced some participants that they were being adequately observed. It was also mitigated by the hybrid program structure because the initial studio-based classes had enabled participants to learn and integrate yoga fundamentals, and to interact face-to-face with their instructor resulting in mutual knowledge, “... having seen us live, she knew what we were capable of ... [so] I trusted when she did push a bit more that it was going to be achievable” (P12). The sense of mastery of core yoga practices and ‘being known’ by the instructor meant that less intense observation and correction online was generally acceptable. However, a few participants suggested they might not work as hard in online classes as a result of less intense observation.

Instructors tended to agree that observation was constrained online,

“I know that if a person was in front of me and I had a 360° view of what was going on in their body I would be able to pinpoint, adjust and correct far better than I can on the screen.” (I1)

Consequently, they adapted their teaching style to address some of the main challenges. For example, painting the studio wall so they could be seen more clearly, asking students to turn so poses could be observed from different angles, more demonstration and step-by-step instruction, encouraging hand signals by the group (thumbs up/down), more time for questions, and the creative use of household items as alternatives to formal props. However, the extra activities resulted in slower progress online which frustrated some of the more advanced participants.

The online platform prevented incidental chat and laughter between group members so this worked best for those with less desire for social connection, but the period of studio-based classes had already established some group camaraderie and the sense of SAGE as a shared experience continued online. Muted classes aided quiet focus but made it hard to communicate with the instructor and so it worked best for people who didn’t want/need to ask questions during classes. Poor visibility of fellow students disadvantaged those who liked to benchmark physical competence or peer check poses.

Despite these barriers to interaction, therapeutic alliance between instructors and our interviewees remained strong, aided by the instructors offering individualised greetings and instruction. Where instructors became aware of problems they also followed up with that person and offered 1:1 consultations which supported some participants who were struggling with an aspect of the program and needed reassurance or modified asanas. However, two disclosed in their interviews that although they were struggling they did not alert their instructor.

We anticipated differences between the classes based on the duration of their studio-based vs online classes (e.g. greater mastery by those in classes with longer studio time, or a more acute sense of loss when it went online?) but none were evident in our data other than the apparent stronger social bonds mentioned earlier in the class with the longest studio time.

## Discussion

This realist process evaluation sought to understand the success of the SAGE yoga trial in maintaining older people’s commitment to a twice-weekly yoga exercise program that started in yoga studios but was forced online by COVID-19 restrictions. We identified six dominant program theories that pivot around 16 causal mechanisms.

We focus next on the implications of these findings for program improvement and scale-up.

### Yoga as a practise for healthy ageing

Our findings highlight the role of context in engagement with the SAGE yoga program, with implications for the program’s promotion. Unsurprisingly, people enrolled in the SAGE trial because they regarded yoga as a suitable method for improving their health or balance (value expectancy) based on prior experiences of yoga or its “good PR”. Previous research has shown that older people tend to perceive yoga as beneficial [13], and an appropriate fall prevention strategy [12], but concerns about pain and exacerbating existing conditions are barriers [14], and many novices have little intention to try yoga [13]. In SAGE, willingness to try yoga was boosted by the trial offering free classes but the participant cohort who took up this offer may have been skewed as the trial implementers found it hard to recruit to the studio in the lowest socioeconomic area which also had a higher proportion of residents from non-English speaking backgrounds. Perceptions of yoga have been shown to be affected by gender, culture and socioeconomic factors [37, 107]. Lastly, SAGE’s exclusive focus on older people was a draw card for our interviewees but some felt their physical limitations or weight made them poor candidates for yoga. These views may be exacerbated by the tendency in popular representations of yoga to depict young, slim, white women in often extreme positions [108]. Promotion of yoga programs should consider methods for addressing misconceptions of yoga, of framing it in culturally appropriate ways, including trialing language-specific classes where appropriate, and use of imagery that helps prospective participants envisage themselves practising yoga.

### Teleyoga as a way forward

Our interviewees greatly valued access to teleyoga during the COVID-19 pandemic lockdown period and, afterwards, were able to attend classes while caring for grandchildren and, in one case, while in hospital. Telehealth interventions have been found effective at promoting physical activity in older people [109] and tend to have high levels of acceptability [110, 111]. Some small studies of teleyoga with this group also indicate feasibility and acceptability [38, 39, 42].

While there are undoubted benefits to online programs and services, there are also challenges, as our analysis confirms. First, telehealth may extend the overall reach of fall prevention and other physical activity programs while disadvantaging certain groups. Technology use is a determinant of health thus lack of digital access or the inability to use it effectively reinforces existing disparities in physical, mental and social health [112, 113]. Older people are a fast growing group of computer and Internet users but they may have less familiarity with technology than younger people and fewer peer opportunities to receive help in using it [92]. Everyone in our interview study had access to an appropriate device and only one person was concerned about the costs of connectivity, but we estimate that most had relatively high socio-economic status, and they all lived in a major metropolitan centre in a country with some of the world’s highest rates of technology ownership and internet usage [114]. Program designers delivering at scale could consider strategies such as free tablet devices when necessary, data cards and the sort of technological support offered by the SAGE team which was well used by participants and yoga instructors. Cost savings in program delivery should be passed on to participants whenever possible to increase access for those on pensions and low incomes.

Second, our interviewees felt teleyoga was less effective at facilitating connection with peers and their instructor. A recent systematic review of group therapy found small decreases in therapeutic alliance in teletherapy but high satisfaction rates despite technical challenges [115]. The authors concluded that teletherapy groups are feasible and produce similar outcomes to in-person treatment. Given that yoga is less focused on interpersonal communication than therapy, it is likely that teleyoga can facilitate sufficient therapeutic alliance. However, effective yoga instruction also requires detailed observation and physical correction of participants’ asanas. The SAGE instructors modified their teaching styles to address some limitations of “2-D” screens and their inability to offer hands-on correction, but they reported mixed feelings about how successful this was. Importantly, half the participants felt videoconferencing hampered observation and correction of poses rendering online classes less efficient, but not unsafe. It would be helpful to collate information across a wider range of yoga instructors to identify techniques that can optimise online yoga, including strategies for building confidence in its effectiveness.

Lastly, teleyoga depends on a suitable home environment. Our interviewees reported a variety of challenges including space constraints, disruption and feelings of intrusion. Matsumoto et al. [36] note that telehealth in a group setting requires reconsideration of participant privacy and consents. This is underscored by a lack of research exploring how features of the home environment influence older peoples’ engagement in physical activity [116]. Where yoga instructors in our study were aware of participant needs and able to identify useful features in their environment, they adapted poses or suggested the use of household items in lieu of the props used for yoga in the studio. This was highly valued by participants who saw it as an example of individualised care as well as a practical intervention, both of which boosted commitment to the program. Considering these findings and their underlying mechanisms (e.g. value expectancy, therapeutic alliance, achievement/mastery, gratitude and accountability), we propose that teleyoga programs for older people adopt a model of 10–15 min one-to-one e-meetings between the instructor and each new participant in which the participant can talk through their goals, capabilities and any concerns; ask questions and introduce the environment in which they will be practicing. Classes that started more recently in the SAGE trial have now adopted this approach. The meeting allows yoga instructors to initiate a personal connection with each participant and to give individualised tips about how to set up for an optimal teleyoga experience, an approach which may have encouraged two of our interviewees who were struggling with asanas to reach out to their instructor. Telehealth is effective when it is person-centred [117] so strategies for enhancing the “human touch” [118] are likely to increase engagement.

### A hybrid yoga program?

Our findings suggest that, where possible, a hybrid model of initial studio-based yoga classes followed by online classes might deliver the best value to participants as it increases access while reducing some of the most pronounced concerns about communication and effective observation and correction in teleyoga. Many older people have a preference for at-home exercise [119], however activities that take people out of their home and offer opportunities for interaction may be more beneficial for ameliorating depression and loneliness, and can kick-start relationships that may be maintained as programs move online. Hybrid telehealth interventions in mental health have been found to be feasible and can be effective [120], while Kaambwa and colleagues’ discrete choice experiment study indicated that older people prefer hybrid models, especially where health facilities are distant from homes [121]. Other telehealth studies suggest patient preferences for initial or periodic face-to-face contact to build rapport and develop confidence in completing treatment exercises effectively [110, 122–124].

However, in weighing up the value of a hybrid model we note that SAGE’s hybrid program may have produced paradoxical findings. On the one hand, interviewees felt that the initial studio-based classes mitigated the impact of decreased interaction and observation online because they had already established relationships in the studio and had learnt and embedded core skills, suggesting the hybrid model is superior to an entirely online model. Yet interviewees did not describe a lack of interaction, observation or correction online (and none felt unsafe in this environment), simply a less effective experience compared to studio-based classes. Therefore, it is possible that studio-based classes set expectations about interactivity, observation and correction that those starting in online classes would not have, especially considering that most interviewees had no prior experience of yoga. Planned follow up with later classes in the SAGE trial which employed a teleyoga model throughout will provide greater insights.

Lastly, although the SAGE program was delivered by different instructors with slightly different teaching styles, our interviewee data did not indicate any discernible difference in how their classes were experienced. This suggests that the program mechanisms were being activated consistently across the whole program. Hawe [125] reminds us that the form or surface appearance of an intervention may vary (and, indeed, is likely to vary) across different intervention sites; however, the intervention retains its integrity (or theoretical fidelity [126]) as long as its function—the underlying mechanisms—are preserved. This approach to intervention fidelity aligns with the increasing popularity of ‘tight-loose-tight’ models of policy and program implementation which address tightly specified problems and set tightly defined targets, but are flexible (loose) in how those targets are reached, thereby recognising variation in implementation sites and enabling creative local delivery methods [127–129].

### Strengths and weaknesses

Realist evaluation’s pragmatic mixed methods approach to data collection is a strength. The use of different ‘lenses of inquiry’ inherent in mixed methods can reveal richer, more comprehensive insights into complex health phenomena [130]. Evaluations of yoga interventions and trials tend to report on levels of adherence and participation satisfaction but seldom attempt to identify the mechanisms that generate these process outcomes (but see Cox & Tylka [97] for a notable exception). Abimbola et al. [131] argue that studies of telehealth seldom differentiate between the message (the service being provided) and the medium (videoconferencing). Our study explored participants’ and instructors’ experiences of the same yoga classes being delivered via two different mediums, providing information about how the medium affected the message.

A weakness in this study was our inability to purposively recruit interviewees from the whole cohort (we were limited to those who had given advance permission to be contacted) or to talk with participants who had withdrawn from the trial. Where reasons for withdrawal were given, they were health-related, but not all withdrawals offered reasons which may indicate undisclosed problems in their engagement with the program. We interviewed yoga program participants soon after they completed the program so recent yoga classes were likely to be fresh in their minds, but they may have had limited recall of earlier classes. Interviewees may have felt some pressure to give socially desirable answers given that the researchers were based in the same research group as the trial designers and implementers Lastly, it is important to consider the context of COVID-19 in interpreting these results: some interviewees suggested that social restrictions may have increased their engagement with SAGE and their acceptance of an online program.

## Conclusion

Our study investigated the transition of a yoga program aimed at preventing falls from studio-based group classes to online classes. The factors that facilitated a successful transition for most participants included the quality of the teaching, the yoga practise itself and the structure of the program. The increased accessibility and convenience of an online class was a benefit for those with transport and health issues but worked less well for those without an appropriate home environment or who had privacy concerns. Many felt that the online interface negatively impacted communication and the effectiveness of yoga instruction compared to face-to-face classes. Using a realist approach we identified causal mechanisms that may have generated these effects. Our results provide useful guidance for the delivery of online yoga classes to people aged 60 years and over.

## Supplementary Information

**Additional file 1.** Research rigour.

## Data Availability

Data sets are qualitative and contain personal identifiable information about participants so are not publically available. We are unable to provide data on request because our ethical approvals state that only the researchers named on this study are able to access the data.

## Abbreviations

SAGE: Successful AGEing yoga trial
ICMO: Intervention activity-Context-Mechanism-Outcome configuration
