# Supplementary material for: What helps older people persevere with yoga classes? A realist process evaluation of a COVID-19-affected yoga program for fall prevention
Source: BMC Public Health. 2022 Mar 8;22:463. doi: 10.1186/s12889-022-12818-5 (PMC8901433; doi:10.1186/s12889-022-12818-5)
Supplement: Supplementary file 1 — Additional file 1. Research rigour. [file 12889_2022_12818_MOESM1_ESM.docx]

## Additional file 1: Research rigour

The tasks we used to strengthen the trustworthiness of this study are described in relation to Ronkainen and Wiltshire’s framework for assessing validity in realist research in sports and exercise psychology, as described below.

**Framework for validity in realist research in sports and exercise psychology**

| **Key questions re validity criteria** | **Tasks for establishing validity** | **Methods used in this study** |
| --- | --- | --- |
| How empirically adequate is the research account? | 1. Establish descriptive validity | Transcription correction  Timely note-taking re emergent theories  Multiple researchers engaged in coding and reviewing accuracy of findings against the data |
|  | 1. Guard against data collection limitations | Purposive sampling for maximum coverage  Multiple methods triangulation  Evolving interview questions taking account of new theories and concepts |
| How ontologically plausible is the research account? | 1. Engage with theoretical explanations of the empirical evidence | Development of tentative initial theories  Theory-checking with interviewees  Continuous exploration of relevant existing theories in the literature |
|  | 1. Take account of context and complexity | In-depth exploration of context from multiple perspectives: diverse participants and intervention deliverers  Particular attention to COVID-19 impacts |
|  | 1. Engage with competing alternative explanations of the evidence | Critical reflexivity  Workshops with ‘critical friends’  Inviting alternative theories from interviewees  Multi-researcher coding  Searching for alternative theories |
| How much practical utility does the research account have? | 1. Findings are used to suggest practical real-world actions | Practical responses, including implications for other yoga programs and scalability, are described in the discussion |
|  | 1. Those practical actions are likely to have a meaningful impact | The theoretical and empirical basis of the trial (which draws on demonstrably successful intervention strategies) plus self-reported benefits from participation and the scalability of the intervention indicate likely impact |

Ronkainen NJ, Wiltshire G: Rethinking validity in qualitative sport and exercise psychology research: a realist perspective. *International Journal of Sport and Exercise Psychology* 2019:1-16.
